# Keratin-Positive Giant Cell-Rich Tumor: A Review and Update

**DOI:** 10.3390/cancers16101940

**Published:** 2024-05-20

**Authors:** Jun Nishio, Shizuhide Nakayama, Kaori Koga, Mikiko Aoki

**Affiliations:** 1 Section of Orthopaedic Surgery, Department of Medicine, Fukuoka Dental College, 2-15-1 Tamura, Sawara-ku, Fukuoka 814-0193, Japan; 2 Department of Orthopaedic Surgery, Faculty of Medicine, Fukuoka University, 7-45-1 Nanakuma, Jonan-ku, Fukuoka 814-0180, Japan; n.shizuhide@gmail.com; 3 Department of Pathology, Faculty of Medicine, Fukuoka University, 7-45-1 Nanakuma, Jonan-ku, Fukuoka 814-0180, Japan; kogakao@fukuoka-u.ac.jp (K.K.); mikikoss@fukuoka-u.ac.jp (M.A.)

**Keywords:** keratin-positive giant cell-rich tumor, xanthogranulomatous epithelial tumor, *HMGA2–NCOR2* fusion, diagnosis, pathogenesis, giant cell tumor of soft tissue, giant cell tumor of bone

## Abstract

**Simple Summary:**

Keratin-positive giant cell-rich tumor (KPGCT) is a recently described mesenchymal tumor characterized by keratin-positive cells and recurrent *HMGA2*–*NCOR2* fusions. Due to the rarity of the disease, KPGCT is poorly understood. Our objective with this review article is to provide a comprehensive reference, offer valuable insights into the clinicopathological characteristics and pathogenesis, and summarize current therapeutic options for KPGCT.

**Abstract:**

Keratin-positive giant cell-rich tumor (KPGCT) is an extremely rare and recently described mesenchymal neoplasm that occurs in both soft tissue and bone, frequently found in young women. It has locally recurrent potential if incompletely excised but low risk for metastasis. KPGCT is histologically similar to conventional giant cell tumors of soft tissue but shows the presence of keratin-positive mononuclear cells. Interestingly, KPGCT also shares some morphological features with xanthogranulomatous epithelial tumors. These two tumors have recently been shown to harbor an *HMGA2*–*NCOR2* fusion, arguing in favor of a single entity. Surgery is the treatment of choice for localized KPGCT. Therapeutic options for advanced or metastatic disease are unknown. This review provides an overview of the current knowledge on the clinical presentation, pathogenesis, histopathology, and treatment of KPGCT. In addition, we will discuss the differential diagnosis of this emerging entity.

## 1. Introduction

Keratin-positive giant cell-rich tumor (KPGCT) is a very rare, low-grade mesenchymal neoplasm first described by Agaimy et al. in 2021 [1]. The authors reported a series of six cases similar to giant cell tumor of soft tissue (GCTST) but with a distinct predilection for appearing in young females, demonstrating keratin expression, and harboring a high mobility group AT-hook 2 (*HMGA2*)–nuclear receptor corepressor 2 (*NCOR2*) fusion. The authors proposed the term “keratin-positive giant cell-rich soft tissue tumor with *HMGA2*–*NCOR2* fusion” for this novel entity. Meanwhile, Panagopoulos et al. demonstrated this *HMGA2*–*NCOR2* fusion in six cases of osteoclastic giant cell-rich tumor of bone [2]. Keratin expression was not mentioned. In 2022, Dehner et al. described a series of nine cases showing overlapping clinical and histological features of KPGCT and xanthogranulomatous epithelial tumor (XGET) [3]. The *HMGA2*–*NCOR2* fusion was identified in seven out of nine cases. The authors suggested that these two tumors might represent morphological variants of a single entity and have uncertain biological potential. Clinically, KPGCT of soft tissue (KPGCTST) frequently occurs in the superficial soft tissue of the extremities, while the axial skeleton is the most common site for KPGCT of bone (KPGCTB) [1,2,3,4,5,6,7,8]. Surgery is the treatment of choice for localized KPGCT. There is currently no consensus regarding the optimal treatment strategy for advanced or metastatic disease. In this article, we review clinical, radiological, histological, cytogenetic, and molecular genetic features of KPGCT and summarize the current management. In addition, we will discuss the differential diagnosis of this recently described entity.

## 2. Clinical Characteristics

KPGCT primarily occurs in younger adults, with a peak incidence age of 20–30 years, and shows a strong female predilection [1,2,3,4,5,6,7,8]. The etiology of this neoplasm in unknown. The majority of KPGCTSTs present as a superficial soft tissue mass or nodule in the extremities. KPGCTST may also occur in less common sites such as the trunk and head and neck [1,4,5]. The diameter ranges from 1.2 to 6.0 cm [1,4,5]. On the other hand, KPGCTB frequently presents with pain localized to the involved anatomical site, with or without swelling [2,6]. Pathological fracture may be present [3]. Although KPGCTB can occur at almost any osseous site, the vertebral body is the most common site to date [6].

Due to the rarity of the disease and a lack of knowledge regarding this condition, the clinical behavior has not been fully elucidated. To the best of our knowledge, fewer than 40 cases of KPGCT have been reported in the English language-based literature [1,2,3,4,5,6,7,8]. In two case series, no recurrences are reported to have been detected in any cases of KPGCTST [3,5]. In contrast, Perret et al. reported that one KPGCTST patient had local recurrence 8 months after the initial fragmented excision and has been disease-free for 16 months after re-excision with negative margins [4]. The authors also described two cases of KPGCTB that developed local recurrences after curettage. Distant metastases are rare in KPGCT, and its malignant transformation has not been described. However, Patton et al. reported one patient with KPGCTB that had local recurrence 5 months after initial presentation and then developed multiple metastatic soft tissue diseases [6]. Based on these findings, we suggest that close clinical and radiological follow-up may be necessary to facilitate early detection of local recurrence and distant metastasis, especially in KPGCTB.

## 3. Imaging Features

There is only very limited description of the imaging appearance of KPGCT. In KPGCTST, radiographs may be normal or reveal a non-specific soft tissue mass without calcification. Radiographically, KPGCTB typically appears as an expansile lytic lesion [4]. The margins may be well or poorly defined. Cortical destruction and soft tissue extension can be seen [3,4,6]. Ultrasonography usually reveals a hypoechoic, heterogeneous mass with slightly lobulated margins in KPGCTST [3]. Color Doppler examination may display hypervascularity. Computed tomography (CT) shows a lytic lesion with soft tissue attenuation in KPGCTB [3,4,6]. Using magnetic resonance imaging (MRI), the lesion shows slightly hyperintense relative to skeletal muscle on T1-weighted images and variable heterogeneous signal intensity on T2-weighted images [1,3,6,9]. Contrast-enhanced MRI demonstrates avid enhancement of the lesion [3].

## 4. Pathogenesis

It has been noted that a subset of osteoclastic giant cell-rich tumors of bone display near-diploid karyotypes with 12q rearrangements [2]. Intriguingly, a balanced translocation t(12;12)(q13–15;q24) has been described as the sole anomaly in a single case. In addition to prominent rearrangements of 12q, the presence of ring chromosomes has been detected in a single case.

KPGCT is genetically characterized by an *HMGA2*–*NCOR2* gene fusion [1,4,5,6,7]. Interestingly, the same fusion has also been identified in XGET and hybrid KPGCT/XGET [3,8], suggesting a pathogenetic link between these entities. The fusion most often involves exon 3 of *HMGA2* and exon 16 of *NCOR2* [1,3,4,5,6]. *HMGA2*, located at 12q14.3, belongs to the HMG protein gene family, which encodes a small non-histone chromatin-associated protein [10]. The *HMGA2* gene is highly expressed in embryonic stem cells during embryogenesis and contains five exons [11]. The first three exons encode the AT-binding domain site, whereas exon 4 encodes a protein linker, and exon 5 encodes the acidic domain, which does not activate transcription. A huge intronic sequence (>140 kb) separates the third from the fourth exon [11]. It is noteworthy that *HMGA2* is also involved in gene fusion in a variety of mesenchymal tumors, including lipoma, uterine leiomyoma, aggressive angiomyxoma, pulmonary chondroid hamartoma, soft tissue chondroma, and dedifferentiated liposarcoma [12,13,14,15,16]. In most cases, *HMGA2* fuses out-of-frame with the 3′-end partner gene or with intergenic sequences [2]. The *HMGA2* fusion protein can both inhibit and activate gene expression by modifying chromatin arrangement. *NCOR2*, located at 12q24.31, encodes a nuclear receptor co-repressor that mediates transcriptional silencing of certain target genes. The *NCOR2* gene is widely expressed in human tissues and contains 45 exons [17]. To date, only a small number of *NCOR2* fusion partners have been identified, including neurotrophic receptor tyrosine kinase 1 (*NTRK1*) and GLI family zinc finger 1 (*GLI1*) [18,19]. Although the oncogenesis of *NCOR2* fusions remains unknown, a de-regulating effect on gene expression through interaction with other transcription factors has been suggested [19]. Additionally, *NCOR2* rearrangements have been reported as a likely incidental finding in a dedifferentiated liposarcoma with mdm2 proto-oncogene (*MDM2*) amplification [20]. Further studies are required to elucidate the exact mechanism of this *HMGA2–NCOR2* fusion protein in KPGCT.

In 2023, Perret et al. reported that whole RNA sequencing showed similar levels of expression of the colony-stimulating factor 1 (CSF1)/CSF1 receptor (CSF1R) axis between KPGCT and tenosynovial giant cell tumor (TSGCT) [4]. Most recently, Dehner et al. identified strong expression of *CSF1* mRNA and high-level *CSF1* gene expression in KPGCT, XGET, and hybrid KPGCT/XGET [8]. These findings indicate that the CSF1/CSF1R pathway is involved in the pathogenesis of KPGCT and suggest that CSF1R inhibitors may have efficacy in the treatment of a subset of advanced/metastatic KPGCTs.

## 5. Histopathology

Grossly, KPGCTST usually appears as a well-circumscribed but unencapsulated or partially encapsulated mass with a tan to brown lobulated cut surface [1,5].

Histologically, KPGCT shows a nodular/multinodular architecture and is composed of bland plump epithelioid or ovoid to spindled mononuclear cells with evenly distributed osteoclast-like giant cells. In addition, foamy macrophages and Touton-type giant cells may be present [4]. A variably abundant lymphoid reaction at the periphery of the lesion can be seen [1]. Small foci of ischemic-type necrosis may be found [1,4]. Stromal hemorrhage and hemosiderin deposition are frequently observed [1,4,5]. Unlike GCTST, metaplastic bone formation and vascular invasion are absent [4,5]. Mitotic activity is generally low, ranging from 3 to 10 mitoses per 10 high power fields (median: 6) [4].

Immunohistochemically, the mononuclear cells are positive for pan-keratin. The osteoclast-like giant cells demonstrate strong positive staining for CD68 but are negative for CD163 [1]. Immunostainings for desmin, SATB homeobox 2 (SATB2), and H3.3 G34W are typically negative [1,4,5]. SWI/SNF-related matrix-associated actin-dependent regulator of chromatin subfamily B member 1 (SMARCB1) expression is retained (normal) [1]. We speculate that immunohistochemistry may play a crucial role in the diagnosis of KPGCT.

## 6. Management

### 6.1. Localized Disease

Surgical excision is the standard treatment for localized KPGCTST. The main goal of surgical therapy is to provide local control of the disease and to secure long-term survival of the patient without reasonable functional and esthetic impairment. The surgical procedure is complete excision with negative margins (R0, no residual microscopic tumor). Re-excision is considered for patients with focally positive margins. It has been suggested that excellent local control in low-grade soft tissue sarcoma (STS) may be achieved with microscopic margins greater than 2 mm [21]. Currently, an adequate margin of excision for KPGCTST is not well established because of its infrequent occurrence and a limited number of reported cases. On the other hand, the surgical procedure selected depends on the extent of the lesion in KPGCTB. The most widely accepted procedure is curettage and filling of the defect with bone graft, bone cement, or bone substitute. As with the treatment of conventional giant cell tumor of bone (GCTB), extensive curettage using a high-speed burr may be performed to reduce the local recurrence rate. En bloc excision may also be indicated, although it may result in loss of function of the joint. Further prospective randomized trials are required to better define optimal treatment approaches for localized KPGCT.

Radiation therapy (RT) can be used as perioperative treatment strategies to improve local disease control. There are currently no reports concerning the use of perioperative RT in patients with KPGCT. In our opinion, RT should be reserved for KPGCT not amenable to surgical excision.

### 6.2. Advanced/Metastatic Disease

The development of unresectable locally advanced or metastatic KPGCT is associated with a worse prognosis. There is currently no regulatory approved treatment for advanced/metastatic KPGCT.

Pexidartinib is an orally available small molecule tyrosine kinase inhibitor with potent and selective activity against the CSF1R [22]. In 2019, the United States Food and Drug Administration (FDA) approved pexidartinib capsules for the treatment of adult patients with symptomatic TSGCT associated with severe morbidity or functional limitations and not amenable to improvement with surgery. Recently, Brahmi et al. reported the use of pexidartinib in the treatment of unresectable TSGCT with an *HMGA2*–*NCOR2* gene fusion [23]. In this case, the patient achieved a compete response still ongoing at 17 months. As mentioned above, the CSF1/CSF1R pathway has been identified in KPGCT. Based on these findings, we speculate that the use of pexidartinib in treating advanced/metastatic KPGCT may show promise.

Denosumab, a fully human monoclonal IgG2 antibody, is widely used to treat unresectable GCTB in adults and skeletally mature adolescents [24,25,26]. It acts by binding to and inhibiting the receptor activator of the nuclear factor kappa B ligand (RANKL), resulting in a loss of osteoclasts. In addition, promising results have been reported regarding the off-label use of denosumab in other giant cell-rich tumors, including aneurysmal bone cyst (ABC) [27,28] and central giant cell granuloma [29,30]. Therefore, it is hypothesized that the use of denosumab may also be beneficial and effective in selected cases of KPGCT. Recently, Perret et al. reported the use of denosumab in a 32-year-old woman with recurrent KPGCTB of the patella [4]. Three months after primary curettage, the patient developed local recurrence and received denosumab therapy for 4 months, followed by surgical excision with focal positive margins (R1). No sign of local recurrence was identified at 6 months after re-surgery. Large prospective clinical trials are required to evaluate the role and also the side effects of denosumab in the treatment of KPGCT.

## 7. Differential Diagnosis

The differential diagnosis for KPGCT is broad and should include benign, intermediate, and malignant giant cell-rich tumors. Notably, KPGCT and XGET show overlapping morphological, immunohistochemical, and molecular genetic features, strongly suggesting that these tumors represent a single entity [1,3,31]. In our experience, KPGCT is most often confused with GCTST and GCTB. The corresponding clinicopathological and molecular characteristics are summarized in Table 1.

XGET, first described by Fritchie et al. in 2020 [31], is an extremely rare, low-grade mesenchymal neoplasm that occurs in both superficial soft tissue and bone. Like KPGCT, it generally occurs in young adults and shows a strong female predilection. The diameter ranges from 2.0 to 7.0 cm [31]. XGET usually reveals a solid mass with soft tissue attenuation on CT, intermediate signal intensity on T1-weighted images, variable heterogeneous signal intensity on T2-weighted images, and avid enhancement following contrast administration [31]. Surgery is the is the treatment of choice for localized XGET. Grossly, XGET generally presents as a circumscribed uninodular mass, often surrounded at least in part by a fibrous capsule. Histologically, XGET consists of sheets of foamy macrophages, Touton-type giant cells, osteoclast-like giant cells, and mixed chronic inflammatory cells. Small foci of necrosis may be seen. Careful inspection, however, discloses small aggregates of bland mononuclear cells, sometimes with a distinctly eosinophilic cytoplasm [32]. Mitotic activity is very low. Immunohistochemically, the mononuclear cells are diffusely positive for pan-keratin. Endothelial and myoid markers are negative, and SMARCB1 expression is retained [31]. Like KPGCT, XGET harbors an *HMGA2*–*NCOR2* gene fusion [3,8]. As mentioned above, *CSF1* gene expression has also been noted in XGET [8]. Interestingly, a pleckstrin homology and RUN domain containing an M1 (*PLEKHM1*) mutation has been identified in a single case [31]. The natural history of XGET appears to be favorable, although the optimal management is not well established.

GCTST, first described by Salm and Sissons in 1972 [33], is a locally aggressive neoplasm of intermediate malignancy that primarily arises in the soft tissue of the upper and lower extremities. Unlike KPGCT, it generally occurs in middle-aged adults and shows no gender predilection [34]. KPGCT typically presents as a usually painless, superficial soft tissue mass. The diameter ranges from 0.7 to 10.0 cm [35,36,37]. Using MRI, GCTST usually reveals a solid soft tissue mass with low to intermediate signal intensity on T1-weighted images, variable signal intensity on T2-weighted images, and avid enhancement following contrast administration [38,39,40]. Positron emission tomography (PET) imaging demonstrates increased fluorodeoxyglucose (FDG) uptake with high standard uptake values [38]. Wide excision appears to be the standard treatment for localized GCTST. RT may be considered for cases with positive surgical margins due to the close proximity of critical structures [41]. Local recurrences occur in 6.2–21% of cases [35,36]. Distant metastases are rare in conventional GCTST. In contrast, high-grade GCTST possesses high metastatic potential [37,42,43]. Grossly, GCTST is a well circumscribed, mostly solid, nodular mass. Gritty regions corresponding to mineralized bone are frequently present. Histologically, GCTST is similar to KPGCT and shows a mixture of round to oval mononuclear cells and osteoclast-like giant cells in a richly vascularized stroma. Unlike KPGCT, metaplastic bone formation is present in about 40–50% of cases. In addition, vascular invasion is seen in up to 50% of cases [35,36]. It should be kept in mind that high-grade GCTST exhibits increased mitotic activity and nuclear pleomorphism, and necrosis is often found. Immunohistochemically, the mononuclear cells are focally positive for CD68 and smooth muscle actin (SMA) but negative for pan-keratin. Unlike KPGCT, GCTST lacks an *HMGA2*–*NCOR2* gene fusion [1]. These findings help to distinguish GCTST from KPGCT. Interestingly, recent molecular studies have shown that GCTST lacks H3.3 histone A (*H3-3A*) gene mutations [44,45].

TSGCT, formerly known as giant cell tumor of tendon sheath or pigmented villonodular synovitis, is a benign mesenchymal neoplasm that most often arises from the synovium of joints, bursae, and tendon sheaths. TSGCT can be divided into localized and diffuse types on the basis of its growth pattern. Malignant TSGCT is exceedingly rare and can arise de novo or following multiple recurrences of conventional TSGCT. Localized TSGCT presents as a slow-growing, painless, well-circumscribed mass or nodule that primarily occurs in the digits, especially the fingers. In contrast, diffuse-type TSGCT is defined as a poorly circumscribed, infiltrative soft tissue mass that usually occurs in large joints, particularly the knee. TSGCT affects all age groups but usually young and middle-aged adults, with a slight female predilection [46]. Using MRI, TSGCT usually shows a soft tissue mass with low to intermediate signal intensity on T1-weighted images, heterogeneous low and high signal intensity on T2-weighted images, and intense heterogeneous enhancement following contrast administration. Surgery is the mainstay of the treatment of TSGCT. In diffuse-type, wide excision, when possible, would be an excellent choice for local control. In 2019, the FDA approved pexidartinib for adult patients with symptomatic TSGCT associated with severe morbidity or functional limitations not amenable to improvement with surgery [47]. Local recurrences are more common in diffuse-type TSGCT and occur in 40–60% of cases [46]. Histologically, TSGCT is composed of an admixture of mononuclear cells, osteoclast-like giant cells, foamy macrophages, and chronic inflammatory cells. There are two types of mononuclear cells: small histiocyte-like cells and larger epithelioid cells. Compared with KPGCT, TSGCT has prominent stromal collagenization. Clusterin expression is found in the large mononuclear cells [48]. Desmin-positive cells are also seen in 43% of cases [49]. The cytogenetic hallmark of TSGCT is the presence of 1p13 rearrangement [50]. The most common translocation is t(1;2)(p13;q37), resulting in a collagen type VI alpha 3 (*COL6A3*)–*CSF1* gene fusion [51]. Actually, only a small subpopulation of cells carries the translocation involving *CSF1*. Surprisingly, recent molecular studies showed that four TSGCT cases harbored the *HMGA2*–*NCOR2* gene fusion [23,52]. It is of interest that one patient with an unresectable diffuse-type TSGCT, lacking the expected *COL6A3*–*CSF1* gene fusion, completely responded to pexidartinib [23]. In this case, keratin expression was not mentioned. We speculate that all these cases may represent KPGCT.

GCTB is an intermediate, locally aggressive, but rarely metastasizing neoplasm. It accounts for 4–5% of all primary bone tumors and typically affects the ends (epiphyses) of long tubular bones [53]. GCTB can occur at any age but has a peak incidence in the third and fourth decades of life, with a slight female predilection. The most commonly involved sites include the distal femur, the proximal tibia, and the distal radius. GCTB presents with pain, swelling, and occasionally restricted joint mobility. Pathological fracture occurs in 5–12% of cases [53]. Malignant transformation of GCTB is relatively uncommon [54]. Similar to KPGCTB, radiographs usually show an eccentric, purely lytic lesion with a well-defined but non-sclerotic margin. The overlying cortex is occasionally destroyed, and the lesion expands to the soft tissue. Periosteal new formation is rarely seen. Using MRI, the lesion usually reveals intermediate or decreased signal intensity on T1-weighted images, low to intermediate signal intensity on T2-weighted images, and heterogeneous enhancement following contrast administration. Fluid–fluid levels are seen in 14% of cases [55]. Surgery is the mainstay of treatment for conventional GCTB. Intralesional curettage strikes a good balance between controlling disease and preserving optimum function in the majority of cases. Various adjuvant therapies, such as ethanol, phenol, hypertonic saline, and liquid nitrogen, have been used to reduce the postoperative recurrence rate. Aggressive GCTB may require wide excision and reconstruction with a modular endoprosthesis. As mentioned above, denosumab is used to treat unresectable or advanced GCTB [24,25,26]. Local recurrences after curettage occur in up to 50% of cases, usually within 2 years [53]. Lung metastases are seen in 3–7% of cases, often following local recurrence [53]. Grossly, GCTB is soft and has a characteristic dark brown color. Histologically, GCTB is composed of an admixture of round to oval mononuclear cells, osteoclast-like giant cells, and foamy macrophages. Unlike KPGCT, reactive/metaplastic bone formation may be present. In addition, mitotic activity, necrosis, and vascular invasion can be observed. In contrast, malignant GCTB shows severe pleomorphism, spindle morphology, and atypical mitotic figures [56]. At least focally, however, areas of conventional GCTB are present in primary malignant GCTB. Importantly, unlike KPGCT, the mononuclear cells are positive for H3.3 G34W [57]. Moreover, the vast majority of GCTBs harbor *H3-3A* gene mutations [58,59,60]. These findings suggest that H3.3 G34W immunohistochemical staining and *H3F3A* mutational testing can be a useful adjunct to differentiate GCTB from KPGCT.

ABC is a benign but locally destructive neoplasm that usually arises in the metaphyses of long tubular bones and the posterior elements of the vertebrae. Rarely, it can affect the soft tissue [61]. ABC can occur at any age but has a peak incidence in the first and second decades of life, with no gender predilection [62]. Pain and swelling are the most common complaints. Radiographically, ABC typically shows a lytic, expansile, usually eccentric lesion with a well-defined margin. CT reveals a well-delineated lytic lesion, usually with a thin surrounding rim of reactive bone. MRI highlights fluid–fluid levels that are highly characteristic of ABC. Contrast-enhanced MRI demonstrates peripheral and septal enhancement of the lesion [63]. Surgery is the mainstay of treatment for ABC. The most widely accepted procedure is curettage with added osteosynthesis whenever needed. Over the past three decades, percutaneous sclerotherapy has become an alternative to surgery in selected cases [64]. The use of denosumab has been shown to be effective as a rescue therapy for controlling ABC [27,28]. Local recurrences after curettage occur in 20–70% of cases [62]. Grossly, ABC shows multiloculated blood-filled, cystic spaces separated by thin, tan-white septa. Solid areas can be present and appear brown and fragile. Histologically, ABC is characterized by multiple blood-filled, cystic spaces separated by fibrous septa. The septa lack an epithelial or endothelial lining and consist of bland spindle-shaped cells and scattered multinucleated osteoclast-like giant cells. The woven bone rimmed by osteoblasts frequently follows the border of the fibrous septa. Mitoses are usually conspicuous; however, atypical mitotic figures are absent, and necrosis is uncommon. Immunohistochemically, the lack of expression of pan-keratin can help distinguish ABC from KPGCT. Importantly, about 70% of primary ABCs harbor ubiquitin-specific peptidase 6 (*USP6*) gene rearrangements [62,65]. The most common translocation, t(16;17)(q22;p13), leads to the fusion of cadherin 11 (*CDH11*) with *USP6* [66]. The translocation results in upregulation of USP6 transcription and is only present in the spindle cell population. It is of interest that *USP6* rearrangements have been described in several benign fibroblastic/myofibroblastic tumors, such as nodular fasciitis, cellular fibroma of tendon sheath, myositis ossificans, and fibro-osseous pseudotumor of digits [67]. We speculate that *USP6* rearrangement testing may be a useful adjunct to differentiate ABC from other giant cell-rich tumors including KPGCT.

## 8. Conclusions and Future Directions

KPGCT is an ultra-rare mesenchymal neoplasm with recurrent but minimal metastatic potential that primarily occurs in young adults. It shows a strong female predilection and can affect both soft tissue and bone. Histologically, KPGCT is similar to GCTST and consists of epithelioid/oval mononuclear cells and osteoclast-like giant cells. Notably, it should be kept in mind that KPGCT lacks metaplastic bone formation. Keratin immunostains highlight the diagnostic mononuclear cells. The genetic hallmark of KPGCT is an *HMGA2*–*NCOR2* gene fusion. Surgery is the mainstay of treatment for localized KPGCT. Pexidartinib may be a promising treatment option for selected patients with advanced/metastatic disease. Long-term follow-up is crucial to monitor for any signs of local recurrence or metastasis. Future studies with randomized clinical rials are required to establish the best management of KPGCT.

## Figures and Tables

**Table 1:** Differential diagnosis of KPGCT.

Entity	Age/Gender	Site	Prognosis	Histopathology	Molecular Features
KPGCT	Limited data: young adults; strong female predominance.	Soft tissue and bone; extremities.	Favorable.	Uninodular lesion; variably abundant lymphoid reaction+, keratin+.	*HMGA2*–*NCOR2* fusions.
XGET	Limited data: young adults; strong female predominance.	Soft tissue and bone; extremities.	Favorable.	Uninodular lesion; peripheral lymphoid reaction+, xanthogranulomatous proliferation+, keratin+.	*HMGA2*–*NCOR2* fusions.
GCTST	Middle-aged adults; no gender predominance.	Soft tissue; extremities.	High local recurrence rates but low metastatic potential.	Multinodular lesion; metaplastic bone formation+, keratin-.	Limited data; no *H3-3A* mutations.
TSGCT	Young and middle-aged adults; slight female predominance.	Soft tissue; fingers (LTSGCT) and knee (DTSGCT).	Varied local recurrence rates but no metastatic potential.	Well-circumscribed lesion (LTSGCT) or poorly circumscribed lesion (DTSGCT); larger epithelioid cells+, clusterin+.	*CSF1* fusions (usually with *COL6A3)*.
GCTB	Young and middle-aged adults; slight female predominance.	Bone; long bones.	High local recurrence rates but low metastatic potential.	Well-defined lesion; reactive/metaplastic bone formation+, H3.3 G34W+.	*H3-3A* mutations.
ABC	Children and adolescents; no gender predominance.	Bone; long bones and vertebrae.	High local recurrence rates but no metastatic potential.	Multiloculated cystic lesion; multiple blood-filled, cystic spaces+, H3.3 G34W-.	*USP6* fusions (usually with *CDH11*).

KPGCT: keratin-positive giant cell-rich tumor; XGET: xanthogranulomatous epithelial tumor; GCTST: giant cell tumor of soft tissue, TSGCT: tenosynovial giant cell tumor; GCTB: giant cell tumor of bone; ABC: aneurysmal bone cyst; LTSGCT: localized tenosynovial giant cell tumor; DTSGCT; diffuse-type tenosynovial giant cell tumor; *HMGA2*: high mobility group AT-hook 2; *NCOR2*: nuclear receptor corepressor 2; *H3-3A*: H3.3 histone A; *CSF1*: colony-stimulating factor 1; *COL6A3*; collagen type VI alpha 3 chain; *USP6*; ubiquitin specific peptidase 6; *CDH11*: cadherin 11.

## Data Availability

Not applicable.
